# Effect of a Health System–Sponsored Mobile App on Perinatal Health Behaviors: Retrospective Cohort Study

**DOI:** 10.2196/17183

**Published:** 2020-07-06

**Authors:** Caroline Cawley, Hannelore Buckenmeyer, Trina Jellison, Joseph B Rinaldi, Keri B Vartanian

**Affiliations:** 1 Wildflower Health San Francisco, CA United States; 2 Women and Children's Institute Providence St Joseph Health Renton, WA United States; 3 Center for Outcomes Research and Education Providence Portland Medical Center Portland, OR United States

**Keywords:** mobile health, perinatal health, health behaviors

## Abstract

**Background:**

Pregnancy mobile apps are becoming increasingly popular, with parents-to-be seeking information related to their pregnancy and their baby through mobile technology. This increase raises the need for prenatal apps with evidence-based content that is personalized and reliable. Previous studies have looked at whether prenatal apps impact health and behavior outcomes among pregnant and postpartum individuals; however, research has been limited.

**Objective:**

The primary objective of this study is to assess whether the use of a health system–sponsored mobile app—Circle by Providence—aimed at providing personalized and reliable health information on pregnancy, postpartum recovery, and infant care is associated with improved health outcomes and increased healthy behaviors and knowledge among users.

**Methods:**

This observational study compared app users and app nonusers using a self-reported survey and electronic medical records. The study took place over 18 months and was conducted at Providence St. Joseph Health in Portland, Oregon. The sample included patients who received prenatal care at one of seven Providence clinics and had a live birth at a Providence hospital. Recruitment occurred on a rolling basis and only those who completed the survey were included. Survey respondents were separated into app users and app nonusers, and survey responses and clinical outcomes were compared across groups using univariate and adjusted multivariate logistic regression.

**Results:**

A total of 567 participants were enrolled in the study—167 in the app user group and 400 in the nonuser group. We found statistically significant differences between the two groups for certain behavior outcomes: subjects who used the app had 75% greater odds of breastfeeding beyond 6 months postpartum (*P*=.012), were less likely to miss prenatal appointments (*P*=.046), and were 50% more likely to exercise 3 or more times a week during pregnancy (*P*=.04). There were no differences in nutritional measures, including whether they took prenatal vitamins, ate 5 fruits or vegetables a day, or drank caffeine. We found no differences in many of the infant care outcomes; however, there was an increase in awareness of “purple crying.” Finally, there were no significant differences in measured clinical health outcomes, including cesarean births, length of hospital stays (in minutes), low birth weight infants, preterm births, small-for-gestational-age births, large-for-gestational-age births, and neonatal intensive care unit stays.

**Conclusions:**

The use of the Circle app, which provides access to personalized and evidence-based health information, was associated with an increase in certain healthy behaviors and health knowledge, although there was no impact on clinical health outcomes. More research is needed to determine the impact of mobile prenatal apps on healthy pregnancies, clinical health outcomes, and infant care.

## Introduction

Demand for prenatal apps has increased over the years, as parents-to-be seek reliable information outside of clinical hours [1]. Lee et al [2] showed that apps related to pregnancy and childcare have become an important resource, particularly among first-time parents. There are currently dozens of available pregnancy apps aimed at providing varying degrees of information to individuals throughout their pregnancy and during the postpartum period.

While mobile pregnancy apps can provide education between prenatal visits, studies have shown that pregnancy apps often lack evidence-based information and localized resources that new parents can trust [2-4]. Other studies have shown that many pregnant individuals are turning to the internet to search for information related to pregnancy symptoms before speaking with their health care provider, sometimes consulting unreliable information [1,5]. Patient-facing technology, including apps, should be tailored to the patient to better engage and educate the patient on key risk factors and other issues related to their pregnancy and birth [6]. Some studies have shown that pregnant patients, especially first-time parents and those at high risk, frequently use mobile apps as a source of information on pregnancy and infant care [2,3,7,8]; however, many of the tools found in pregnancy apps have not been shown to be effective [9]. For example, breastfeeding trackers are increasingly prevalent, but very few have been shown to provide evidence-based information and recommendations [9,10]

To find a way to help pregnant patients outside of traditional prenatal appointments, the Providence St. Joseph Health’s Consumer Innovation Team had discussions with 10 providers, including perinatal providers, service line leaders, and educators from the Swedish Medical Center and Providence Health & Services. They also conducted one-on-one interviews with 11 individuals in Seattle, Washington, and 7 individuals in Portland, Oregon, who were either pregnant or new parents. The provider and consumer sessions were facilitated by a 3rd-party user experience research firm. Providers reported that they were limited by time constraints during inpatient visits to address all patient questions, as well as their clinical and nonclinical needs. Pregnant individuals and new parents expressed a desire for information they could trust, that helped them feel “normal,” could be personalized to their unique needs, and that connected them to care quickly when they needed additional support. Based on these discussions, the team designed the Circle app with the following goals:

- Provide Providence and its patients and their families with the opportunity to connect and receive personalized health information to promote healthy pregnancies, births, and pediatric care from birth to 18 years of age;
- Give users access to relevant, evidence-based pregnancy and infant care information they can trust;
- Connect users to prenatal and postnatal care and services;
- Provide informative content, to-do lists, and reminders related to prenatal and pediatric care, including tools to track fetal movement, pregnancy weight gain, vaccines, feeding, and diapers that parents-to-be and current parents can use in consultation with their provider;
- Improve patient retention with Providence St. Joseph Health providers;
- Positively impact family well-being;
- Integrate with Providence’s MyChart and promote Express Care Virtual, which are online services offered by Providence that are aimed at increasing access to care.

Circle launched in 2016 in Portland, Oregon, and the greater Seattle, Washington, area. In 2018, Circle was acquired by Wildflower Health, a digital health company based in San Francisco. As of September 2018, the app is now available to patients across all Providence St. Joseph Health locations. As of November 2019, there were over 45,000 registered Circle users.

This study examined whether use of the Circle app during the prenatal period was associated with improved health and health behavior outcomes among pregnant and postpartum parents. We hypothesize that use of the Circle app would be associated with improved healthy behaviors and knowledge during and after pregnancy and improved clinical outcomes compared to non-Circle app users.

## Methods

### Study Overview

This was an observational study that used surveys and electronic medical records to examine a variety of outcomes on health knowledge, healthy behaviors for pregnancy and infant care, and clinical outcomes. The Providence Health and Services Institutional Review Board approved this study.

### Data Sources

There were two main data sources for this study: the Providence Birth and Infant Care Survey and electronic medical records. The Providence Birth and Infant Care Survey was developed by Providence St. Joseph Health’s Center for Outcomes Research and Education (CORE) in partnership with the Consumer Innovation Team. The survey included validated measures of certain health behaviors and knowledge in the prenatal and postnatal periods as well as infant care. The survey also measured demographics and the use of resources and technology (including the Circle app) to obtain information on pregnancy and infant care.

Electronic medical records from Epic, an electronic health record software application used by Providence St. Joseph Health, included individual-level data on clinic visits, medical procedures, and diagnoses during pregnancy and at birth. These data were used to identify the study sample based on engagement in prenatal care at one of the seven selected clinics and having a live birth at a Providence hospital. This data source was also used to measure clinical outcomes at birth for the birth parent and infant.

### Participants and Recruitment

We used Epic medical health records to select potential study participants based on the following criteria: they had four or more prenatal encounters at one of the seven selected Providence clinics in Portland, were over 18 years of age, and gave birth to a live infant at a Providence hospital in the past 4-6 months. Individuals were excluded if they were an employee of Providence. Recruitment into the study occurred from March 2018 to January 2019. During the study window, a total of 1500 people were identified as potential participants.

Paper surveys were sent out to all potential participants through the mail with a small monetary compensation for their time. Nonresponders received a second survey in the mail as well as an email reminder and electronic versions of the survey. A total of 618 viable surveys were returned for an overall response rate of 41.2%.

Survey responses were used to define the app user group and the comparison nonuser group. The app user group comprised individuals who indicated that they used the Circle app during the prenatal period and the nonuser group comprised individuals who indicated that they did not use the app during the prenatal period or did not use the app at all. Respondents were excluded from the analysis if they did not indicate use of any resources in the prenatal period (n=31) and if their responses concerning Circle use were contradictory (ie, it was unclear if they were, in fact, a Circle user; n=20). After exclusion, the total number of individuals with viable surveys was 567. A total of 167 people qualified for the app user group (those who used Circle in the prenatal period), and 400 people qualified for the comparison nonuser group (those who did not use Circle in the prenatal period).

### Outcome Measures

The Providence Birth and Infant Care Survey includes many validated and developed measures of pre- and postnatal health, health care, health behaviors, and health knowledge. We selected three main types of outcome measures for this study:

- Prenatal care and behavior: patient’s prenatal and postpartum care, including missed appointments; dental care; exercise, prenatal vitamins, fruit/vegetable and caffeine consumption [11-16]
- Infant care: breastfeeding, preparedness for appointment, awareness of “The Period of Purple Crying” (a time early in an infant’s life when there is more crying than normal), and vaccine hesitancy [12,17-20]
- Demographics and socioeconomic status: age, race/ethnicity, gender identity, number of children, marital status, income, education, and insurance type [12]

Clinical outcomes were defined as binary variables (present or absent) using a combination of the International Statistical Classification of Diseases and Related Health Problems-10, Current Procedural Terminology / Healthcare Common Procedure Coding System, and revenue codes. Clinical outcomes included caesarian section, preterm birth (fewer than 37 weeks of gestation), low birth weight (less than 2500 g), small-for-gestational-age birth (weight below the 10th percentile for the gestational age), large-for-gestational-age birth (weight above the 90th percentile for that gestational age), length of stay for a birth event, and presence of a neonatal intensive care unit (NICU) stay.

### Analysis

Univariate logistic regressions were used to identify demographic differences between the app user and nonuser groups. Survey outcome measures with Likert-type scales were collapsed into binary responses for positive and negative responses. For responses on a 5-point Likert scale, we collapsed only the clearly positive responses into the positive group with the remaining responses allocated to the negative group. We did not adjust our significance level based on multiple comparisons, which should be considered in the interpretation of results. Analysis of outcomes were conducted using one-step multivariable logistic regressions constructed with adjusting variables based on significant difference across the two groups and understanding of factors that can impact pregnancy outcomes—age (continuous), race, gross household income, number of children, and insurance type. All analyses were conducted in R, version 3.3.3. We considered *P*<.05 to be statistically significant for the purposes of this study.

## Results

### Demographic and Socioeconomic Profile

The Providence Birth and Infant Care Survey asked questions on demographics and the socioeconomic status of respondents to better understand the profile of individuals using Circle in the prenatal period compared to those who did not. Our results found that the prenatal Circle users and non-Circle users were similar in age and marital status (Table 1). We did find a statistically significant difference in race and ethnicity between users and nonusers (*P*=.01). Circle users were more likely to be white than non-Circle users. Circle users were also more likely to be pregnant with their first child compared to non-Circle users (*P*<.001).

The survey looked at several socioeconomic factors, including self-reported education, household income, and insurance status (Table 2). We found statistically significant differences between the groups in gross household income and insurance type. There were fewer low-income prenatal Circle users than non-Circle users (*P=.*001) and, likewise, fewer Circle users on Medicaid (*P*=.02). Despite this difference in health insurance coverage, there was a similar percentage of people in each group covered by the Providence Health Plan. These differences were adjusted for in our analyses.

**Table 1:** Demographic profile of study participants (N=567). Italicized *P* values are significant.

Characteristic		Prenatal Circle use (n=167), n (%)	No prenatal Circle use (n=400), n (%)	*P* value
**Age group**				.81
	Under 25	7 (4.2)	26 (6.5)	
	25-29	34 (20.4)	84 (21.0)	
	30-34	62 (37.1)	133 (35.3)	
	35-39	53 (31.7)	119 (29.8)	
	40 and older	11 (6.6)	38 (7.5)	
**Race/ethnicity**				*.01*
	White	117 (70.3)	243 (60.7)	
	Hispanic	7 (4.2)	39 (9.8)	
	Asian	14 (8.5)	60 (15.0)	
	Other	29 (17.0)	58 (14.5)	
**Number of children**				
	First child	109 (65.1)	193 (48.4)	*<.001*
	More than 1 child	58 (34.9)	207 (51.7)	
**Marital status**				.65
	Married or domestic partnership	145 (86.6)	341 (85.2)	
	Single, never married	16 (9.8)	49 (12.3)	
	Divorced	2 (1.2)	5 (1.3)	
	Something else	4 (2.4)	5 (1.3)	

**Table 2:** Socioeconomic profile of study participants (N=567). Italicized *P* values are significant.

Characteristic		Prenatal Circle use (n=167), n (%)	No prenatal Circle use (n=400), n (%)	*P* value
**Education**				.10
	High school or less	18 (10.4)	64 (16.1)	
	Some college	23 (14.0)	65 (16.4)	
	Vocational training or 2-year degree	8 (4.9)	27 (7.2)	
	A 4-year college degree or more	118 (70.7)	241 (60.4)	
**Gross household income ($ US)**				*.001*
	$30,000 or less	25 (14.7)	97 (24.3)	
	$30,001 to $50,000	14 (8.6)	66 (16.4)	
	$50,001 to $70,000	22 (12.9)	52 (13.0)	
	$70,001 to $100,000	38 (22.7)	55 (13.8)	
	$100,001 to $150,000	33 (19.63)	75 (18.7)	
**Insurance type**				*.02*
	Private coverage	126 (75.2)	253 (63.3)	
	Medicaid/Oregon Health Plan	21 (12.7)	87 (21.8)	
	I don't have insurance now	4 (2.4)	19 (4.8)	
	Other	16 (9.7)	40 (10.0)	
**Insured by** **Providence Health Plan**				.45
	Yes	70 (41.8)	167 (41.7)	
	No	88 (52.7)	199 (49.8)	
	I don't know	9 (5.5)	34 (8.4)	

### Healthy Behaviors

The study examined prenatal care and healthy habits during pregnancy for the study population (Table 3). We found that very few people in either group felt they were unprepared for their prenatal care appointments and a similar percentage of people in each group received dental care during their pregnancy. We did find that Circle users were significantly less likely to miss prenatal appointments compared to non-Circle users, showing a 45% reduction in the odds of having a missed prenatal care appointment when using Circle (*P*=.046). Survey results also found that Circle users had 50% greater odds of reporting that they exercised three times a week during pregnancy (*P*=.04). All other behaviors measured showed no significant difference.

**Table 3:** Health behaviors of study participants while pregnant (n=541^a^). Italicized *P* values are significant.

Behavior		Prenatal Circle use (n=162), n (%)	No prenatal Circle use (n=379), n (%)	Logistic regression	
				aOR^b^	*P* value
**Prenatal care**					
	Felt unprepared for appointments	10 (6.3)	16 (4.8)	1.24	.62
	Missed prenatal appointments	18 (11.2)	69 (18.1)	0.55	*.046*
	Received dental care during pregnancy	103 (63.7)	215 (56.6)	1.10	.64
**Health behaviors**					
	Exercised 3 times a week	82 (50.6)	147 (38.9)	1.50	*.04*
	Did not take recommended vitamins	8 (4.9)	23 (6.1)	0.91	.84
	Did not eat 5 fruit/vegetables a day	30 (18.8)	79 (20.9)	0.95	.84
	Drank more than 2 caffeinated beverages a day	12 (7.4)	31 (8.2)	1.32	.47

^a^Study participants with responses to health behavior questions.

^b^aOR: adjusted odds ratio. Adjusted for age (continuous), race, gross household income, number of children, and insurance type.

### Caring for Infants

We assessed several aspects of infant care in the survey (Table 4). The results showed a 75% increase in the odds of breastfeeding for 6 months or more among Circle users (*P*=.012). In addition, there was a statistically significant increase in the awareness of the “Purple Crying” period among prenatal Circle users compared to non-Circle users (*P*=.04). We did not observe any differences in preparedness for infant appointments and vaccine hesitancy between groups.

**Table 4:** Responses from participants on caring for infants (n=541^a^). Italicized *P* values are significant.

Response	Prenatal Circle use (n=162), n (%)	No prenatal Circle use (n=379), n (%)	Logistic regression	
			aOR^b^	*P* value
Breastfed for more than 6 months	118 (73.1)	226 (59.6)	1.75	*.01*
Unprepared for infant appointments	4 (2.5)	11 (3.0)	0.50	.28
High vaccine hesitancy	9 (5.6)	31 (8.2)	0.73	.44
Never heard of “Purple Crying”	23 (14.1)	105 (27.8)	0.55	*.04*

^a^Study participants with responses to infant care questions.

^b^aOR: adjusted odds ratio. Adjusted for age (continuous), race, gross household income, number of children, and insurance type.

### Clinical Health Outcomes

We examined clinical health outcomes for prenatal Circle users and non-Circle users, including cesarean births, length of hospital stays (in minutes), infants with low birth weight, preterm births, small-for-gestational-age births, large-for-gestational-age births, and NICU stays. There were no significant differences between the app user and nonuser groups for all measured clinical outcomes (Table 5).

**Table 5:** Clinical outcomes for birth parent and infant (n=541^a^). Italicized *P* values are significant.

Outcome		Prenatal Circle use (n=162), n (%)	No prenatal Circle use (n=379), n (%)	Logistic regression		
				aDiff^b^	aOR^c^	*P* value
**Maternal outcomes**						
	Cesarean delivery, n (%)	40 (24.7)	99 (26.1)	—^d^	0.96	0.857
	Length of stay (minutes), average (SD)	4256 (2615)	4196 (3591)	284	—	0.369
**Infant outcomes, n (%)**						
	Low birth weight	2 (1.2)	3 (0.8)	—	0.82	0.831
	Preterm birth	8 (4.9)	6 (1.6)	—	3.08	0.061
	Small for gestational age	7 (4.3)	10 (2.6)	—	2.90	0.077
	Large for gestational age	3 (1.9)	6 (1.9)	—	1.00	0.997
	NICU^e^ stay	8 (4.9)	20 (5.0)	—	0.72	0.452

^a^Study participants with clinical outcomes data.

^b^aDiff: adjusted means difference. Difference adjusted for age (continuous), race, gross household income, number of children, and insurance type.

^c^aOR: adjusted odds ratio. Odds ratio adjusted for age (continuous), race, gross household income, number of children, and insurance type.

^d^Not applicable.

^e^NICU: neonatal intensive care unit.

## Discussion

### Principal Findings

The Circle app was created with the goal of providing reliable, personalized content to its pregnant patients and new parents. The Providence health system saw the app as a potential tool to better reach patients outside of clinic hours and to help reduce the incidence of negative prenatal and birth outcomes. Data from the self-reported survey demonstrated that the app was associated with improvement in some healthy behaviors—increased exercise during the prenatal period, increased duration of breastfeeding, increased knowledge of the “Purple Crying” period, and a decrease in missed prenatal visits. These findings are in line with research demonstrating the feasibility and suitability of mobile apps in impacting behavioral change in other fields of health [21].

However, this study showed that use of the app was not associated with differences in clinical health outcomes such as premature births, cesarean births, low birth weight babies, and neonatal intensive care unit stays. Previous studies in the mobile health field have also demonstrated the difficulty in impacting clinical health outcomes [10,22,23]. Even in randomized controlled trials, studies that successfully demonstrate the effectiveness of prenatal apps in improving health behavior often fail to find statistically significant differences in neonatal outcomes, delivery, or pregnancy complications [24]. Due to small study populations, many trials may be underpowered to detect clinically and/or statistically significant changes in prenatal health outcomes [25]. More research is needed to better understand how digital health and mobile apps can help move the dial on adverse clinical health outcomes.

Our results contribute to the evolving health care field and the move toward digital care to better engage patients outside of a clinical setting. The use and availability of mobile apps in prenatal care is growing [1,7], and our study shows that the use of prenatal apps that provide reliable, personalized content to the patient can improve some behavior health outcomes among pregnant and new parents.

### Limitations

This study has several notable limitations. First, our study population was limited to individuals who received care at 7 Providence clinics and gave birth at Providence hospitals in Portland, Oregon, limiting the number of people who would be eligible for the survey and creating a bias in the sample. Second, we did not use a random sample and could only include individuals who responded to our survey—to ensure they were or were not Circle users—which is another source of potential bias in our sample. Third, this was an observational study, not a randomized controlled trial (ie, respondents were not randomized to the app user and nonuser groups), meaning that there may be underlying differences between the two groups. Fourth, most outcomes were survey-based, which could be subject to reporting bias, such as individuals overreporting positive behaviors and underreporting unhealthy ones. However, since both groups are self-reporting, we can expect to see the same level of inflation in both groups. Findings from the survey on demographics and socioeconomic status suggest that Circle did not reach a diverse racial or socioeconomic population, limiting the generalizability of this study to other patient populations. Finally, while our survey results indicate that some individuals in the nonuser group were using other app-based resources, we did not make any formal comparisons on whether the Circle app led to different outcomes compared to other apps.

### Conclusion

Our study demonstrated that using Circle was associated with positive impacts on several key health behavior and knowledge outcomes but has no significant impact on clinical health outcomes. These health behaviors and knowledge outcomes have important benefits for healthy pregnancies and healthy infant care. Improvements could be made to target a more diverse audience to promote health equity, to expand the impact on health behaviors and knowledge, and to adjust content and other key features to impact clinical health outcomes. While our results demonstrate the potential utility of using apps to promote healthier behaviors and knowledge, more research is needed to determine the efficacy of health system–sponsored, personalized mobile pregnancy apps and their ability to improve health outcomes among pregnant and postpartum parents and their infants.

## Abbreviations

aDiff: adjusted means difference
aOR: adjusted odds ratio
CORE: Center for Outcomes Research and Education
NICU: neonatal intensive care unit
